# Improving the reproducibility of mixing-time experiments in stirred single-use bioreactors

**DOI:** 10.1007/s00253-026-13931-w

**Published:** 2026-06-29

**Authors:** Isabelle Barth, Christian Holbeck, Charlotte Capitain, Tanja Schirmeister, Percy Kampeis

**Affiliations:** 1 Institute for Biotechnical Process Design, Environmental Campus Birkenfeld, Trier University of Applied Sciences, Campusallee 9913, 55768 Hoppstädten-Weiersbach, Germany; 2 Institute of Pharmaceutical and Biomedical Sciences, Johannes Gutenberg University Mainz, Mainz, Germany

**Keywords:** Single-use bioreactor, Stirred-tank bioreactor, Mixing time, 3D printing, Automation

## Abstract

**Abstract:**

Mixing time is a key metric for characterizing bioreactors, as it governs bulk homogenization and correlates with the underlying flow field and shear exposure. Having knowledge of turbulent flow regimes is particularly important for shear-sensitive (mammalian) cells, which are often cultivated in single-use systems. To characterize such systems in terms of their mixing-time behavior, colorimetric pH-shift assays can be employed. However, operator-dependent variability during the reagent addition often limits the comparability of such experiments. To address this limitation and enhance reproducibility, a compact linear actuator was developed that enables automation and, thus, standardization of the addition process. The device was designed using computer-aided design software, fabricated via 3D printing, and subsequently integrated into mixing-time experiments. It is compatible with both stirred single-use bioreactors and glass bioreactors. For automated experiments using the linear actuator, neither the mixing time nor the replicate variance increased in the single-use bioreactor relative to the glass bioreactor under non-aerated conditions, demonstrating strong comparability between the two systems. The use of the linear actuator increased measurement precision, as evidenced by a 13.6% reduction in the coefficient of variation. This improvement resulted from the injection profiles, which were consistent in their immersion depth, immersion angle, tracer volume, and injection speed. Accordingly, this approach provides a low-cost, plug-and-play method to improve the reproducibility of mixing-time experiments, thereby facilitating bioreactor characterization, technology transfer, and validation of computational fluid dynamics models.

**Key points:**

*Automated acid/base dosing improves reproducibility versus manual addition.*

*Variation of the local mixing is detectable through spatially resolved analysis.*

*Enables robust characterization of both stirred single-use and glass bioreactors.*

**Graphical abstract:**

**Figure:**
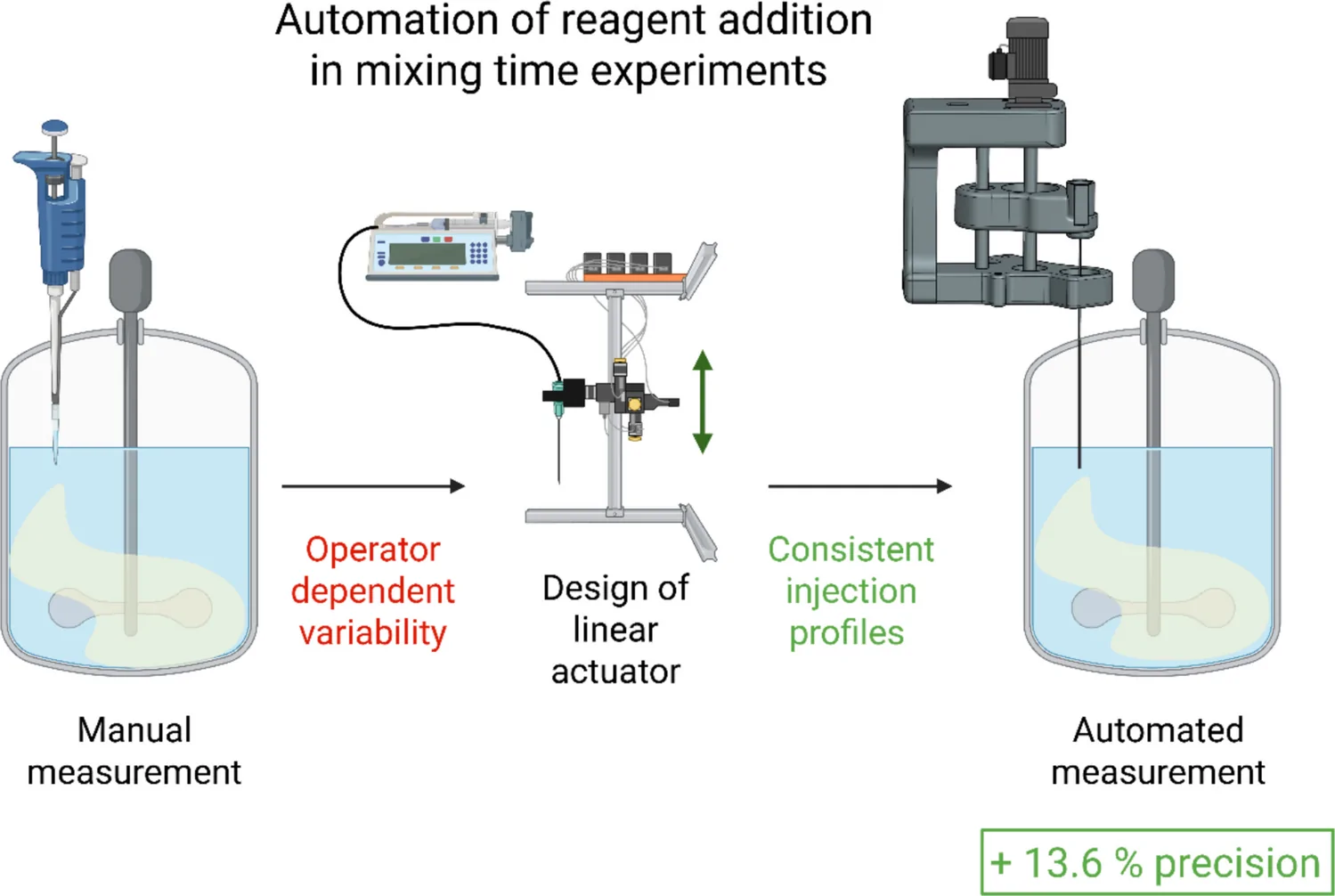

## Introduction

The efficient cultivation of microorganisms requires adequate supplies of oxygen and substrates. To avoid localized nutrient or gas depletion, the broth must be maintained in a homogeneous state inside the bioreactor during cultivation. Mixing time is a practical descriptor of bulk homogenization quality, with shorter mixing times generally reflecting a more uniform distribution of dissolved gases and nutrients within the reaction vessel. Operationally, mixing times can be tuned by adjusting agitation parameters, including the stirrer’s rotational speed and geometry (e.g., using a Rushton turbine or a three-bladed segment impeller). However, the hydrodynamic conditions that shorten mixing times also influence shear stresses and the overall flow field. Although it reduces the mixing time, excessive agitation can generate shear and turbulent eddies that exceed the tolerance of shear-sensitive cells, such as mammalian cells or filamentous fungi, resulting in cellular damage or lysis (Soerjawinata et al. 2021; Fitschen 2022). Consequently, mixing optimization must balance rapid macromixing with acceptable shear exposure.

The degree of mixing (homogenization) in a bioreactor is typically quantified using the dimensionless local concentration *θ*(*r*,*t*), which characterizes the state of mixing at position *r* and time *t* (see Eq. 1). The mixing time is generally defined as the time required for the deviation of the dimensionless local concentration *θ*(*r*,*t*) from its final value to fall within a specified tolerance *ε*. A tolerance of *ε* = 0.01 is problematic because true inhomogeneities often cannot be distinguished from measurement noise. In contrast, a tolerance of *ε* = 0.1 allows deviations up to 10%, which is often considered too imprecise. A value of *ε* = 0.05 represents a suitable compromise between ensuring sufficient homogeneity and maintaining a realistic measurement accuracy. Therefore, this criterion was chosen for this study, and the mixing time *t*_95_ has been defined as the point in time at which the condition |θ(*r*,*t*) − 1|≤ 0.05 is satisfied. 

1 $$\theta\left(r,t\right)=\frac{c\left(r,t\right)-c\left(0\right)}{c\left(\infty\right)-c\left(0\right)}$$

where *θ*(*r*,*t*) is the dimensionless local concentration at position *r* and time *t*; *c*(*r*,*t*) is the measured value (e.g., concentration, pH value, color) at position *r* and time *t*; *c*(0) is the measured value (e.g., concentration, pH value, color) at the start; and *c*(∞) is the steady-state final value (e.g., concentration, pH value, color).

The mixing performance can be observed visually, for example, through the change in color of a pH-sensitive tracer. The use of an appropriate decolorization method to measure mixing times was first proposed by Fox and Gex (1956). The mixing performance can be uneven throughout the reaction vessel, resulting in unmixed areas. In such cases, the local mixing time represents the time required for the complete homogenization at a specific point within the reaction vessel, reflecting spatial variations in mixing efficiency. In severe cases, the terminal blending time is achieved only through diffusion (Godleski and Smith 1962). To remove the subjectivity inherent in estimating time by the naked eye, Cabaret et al. (2007) employed a digital camera to record the decolorization process in a transparent stirred tank. Numerical simulations using computational fluid dynamics (CFD) are widely used to quantify mixing in stirred tanks (Sahu et al. 1999; Zhang et al. 2009; Wutz et al. 2020); however, these simulations must be validated experimentally (Rautenbach et al. 2026). Fitschen et al. (2021) used a camera-based method to validate numerical simulations (i.e., the Lattice-Boltzmann method), showing good agreement between the simulations and the corresponding experiments.

The conventional step-by-step approach for determining local and global mixing times, which employs a camera-based, colorimetric pH-shift assay, is as follows (Fitschen et al. 2021): Acid is injected into an alkaline liquid containing the pH indicator bromothymol blue. As the reagent is dispersed, local color changes occur, and these are recorded on video. A subsequent image analysis determines the time from reagent addition to complete homogenization, which is defined as the full color transition from blue to yellow. This method offers greater insight into the mixing process and can be used to determine local mixing times by observing the color changes in specific areas. In principle, local mixing can be evaluated for small-volume regions within each pixel of the camera image. This makes it possible, for example, to identify stagnant (“dead”) zones or to characterize the effect of different stirrers. The workflow supports spatially resolved analyses through region-of-interest (ROI) definitions and provides time-stamped images of intermediate states. This method also provides 2D color-coded diagrams showing local mixing times throughout the reaction vessel. The advantages of this technique include near-instantaneous indicator response (minimal measurement lag), full-field visualization for the simplified detection of dead zones, and a lower cost and risk compared with laser-based diagnostics.

Since the early 2000 s, single-use (SU) technologies have been increasingly utilized for biopharmaceutical processes. SU technologies have grown rapidly since the first wave-induced bioreactor in 1998, and the first SU stirred tank in 2004 (Shukla and Gottschalk 2013; Eibl and Eibl 2019). These technologies offer several advantages, including a reduced workload for preparation, cleaning, sterilization, and validation; lower water and energy consumption; a reduced risk of cross-contamination; and greater process flexibility (Klutz et al. 2015). These benefits make SU bioreactors highly attractive for GMP operations and multi-product facilities. In the biopharmaceutical industry, cross-contamination is a key concern, particularly when spore-forming microorganisms are involved (Sandle 2016). One commonly used SU bioreactor is the Univessel® SU (Sartorius Stedim Biotech GmbH), which is equipped with a two-stage stirrer featuring three-bladed segment impellers. This bioreactor has been applied to stem cell production and CHO cell cultivation (van den Bos et al. 2013; Schirmaier et al. 2014) as well as the cultivation of fungi (Soerjawinata et al. 2021), plant cells (Lehmann et al. 2014), microalgae (Lehmann et al. 2013), bacteria (Dreher et al. 2013), and yeast (Mikola et al. 2007).

Although CFD studies can also be utilized to develop and optimize SU bioreactors, assessing the accuracy of the CFD model requires high-quality validation data. Consequently, the experimental generation of standardized and reproducible parameters, such as mixing time, is critically important. The existing literature highlights various methods for mixing-time experiments, which differ in terms of tracer injection speed and position as well as in monitoring position (Hartmann et al. 2006). In their comparability study, Kraume and Zehner (2001) reported a scattering of 10% to 20% in the dimensionless mixing time, regardless of the method used. These authors also found that the fixed position for the injection played a crucial role in preventing further deviations.

Although the optical approach described above has been successfully applied to glass bioreactors (Bartczak and Pilarek 2023), challenges arise when characterizing SU bioreactors. Compared with highly transparent (double-walled) glass vessels, SU bioreactors made of plastic are less transparent (i.e., they appear “milky”), which makes color evaluation more difficult. Consequently, optical analyses in SU bioreactors are subject to considerable inaccuracies. Additionally, SU bioreactors typically use opaque heating/cooling jackets that further obstruct direct visual access. In the present study, a camera-based method with an automated evaluation was applied to stirred SU bioreactors to extract the global and local mixing times from video recordings. The objective of this study was to overcome the aforementioned challenges and to establish a highly reproducible method for determining mixing times in SU bioreactors.

## Materials and methods

With respect to the tolerance criterion, the mixing time *t*_95_ was employed and defined as the time at which the condition |*θ*(*r*,*t*) − 1|≤ 0.05 was satisfied. The mixing-time experiments were performed using a colorimetric pH-shift method with bromothymol blue as an indicator (Lamberto et al. 1996; Gaugler et al. 2023). In this method, video recordings were captured with a camera system and evaluated using a Python-based script to determine the global or local mixing times *t*_95_ (Barth et al. 2026). The camera system consisted of a Nikon D7500 camera (Nikon Corp.) with a Zeiss Milvus 2.0/50M ZF.2 lens (Carl Zeiss AG). The videos were recorded with a spatial resolution of 1920 × 1080 px^2^ and a temporal resolution of 60 Hz. In the setup beneath the dark tent, the exposure time was set to 1/160 s, and the aperture was set to F8. The camera settings and ISO sensitivity were adjusted so that none of the indicator’s color states was overexposed or underexposed. For comparison purposes, tests were carried out in both a glass bioreactor (see “Experimental setup with glass bioreactor”) and a stirred SU bioreactor (see “Experimental setup with single-use bioreactor”).

To accurately measure the time interval during which the mixing takes place, it is essential to determine the starting point of the mixing-time experiment. For this purpose, the start time was synchronized via a step change in the brightness of an LED backlight. To further improve reproducibility, particularly among different operators, the addition of tracer (i.e., acid) and activation of the backlight were automated (see “Automated addition of acid for mixing-time experiments”). To compare the reproducibility of the automated mixing-time experiments, the acid was manually added using an Eppendorf Research® mechanical pipette (Eppendorf Vertrieb Deutschland GmbH). The manual measurements were performed by eight different operators, and all experiments were conducted in triplicate for each operator and stirrer tip speed setting.

### Experimental setup with glass bioreactor

**Fig. 1:**
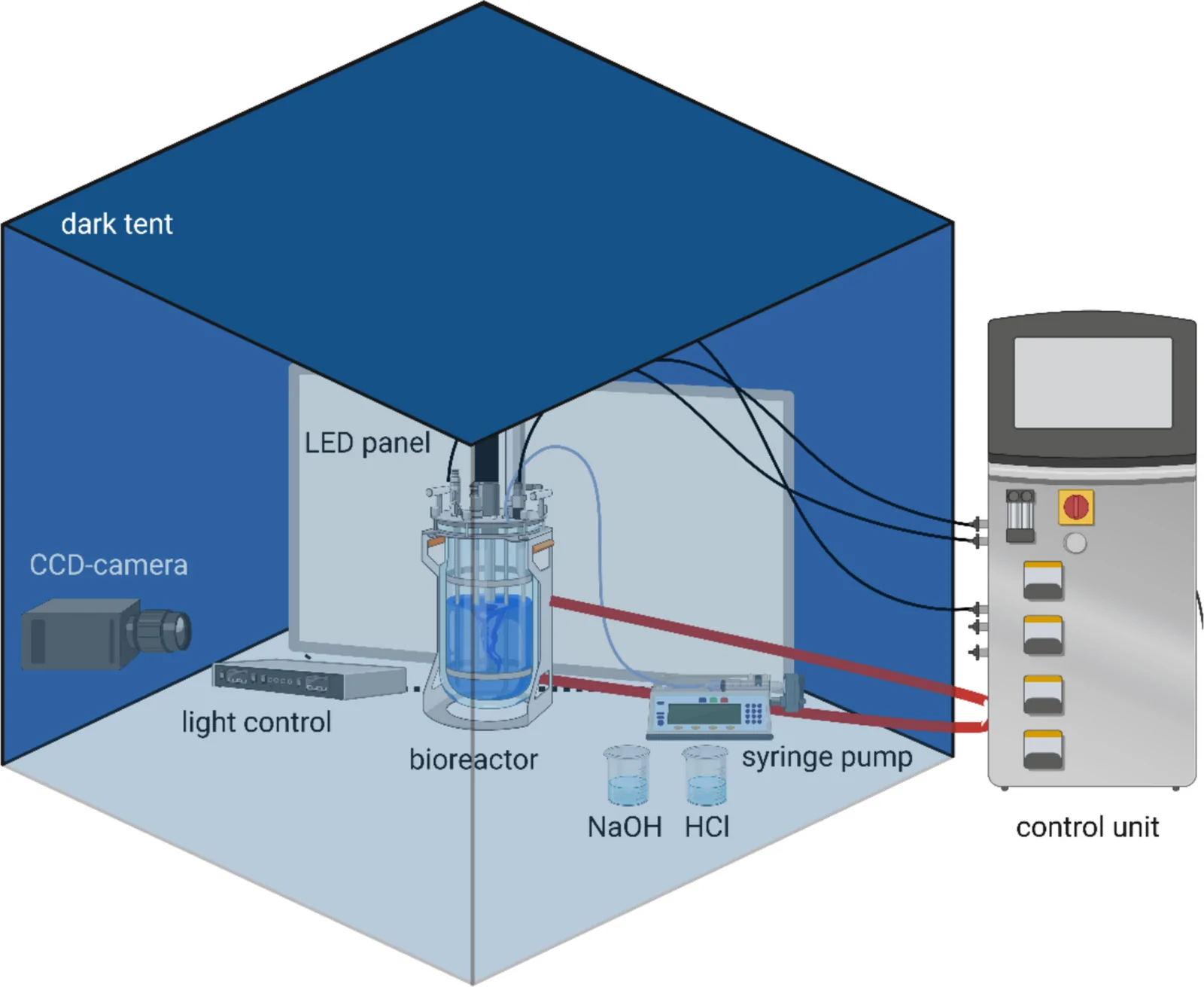
Setup of the mixing-time experiments in a dark tent with the bioreactor positioned between a video camera and an LED panel for backlight illumination (Barth, I. (2026) https://BioRender.com/3aqrteq)

For this work, a Univessel® 2 L DW (double-walled) glass bioreactor (Sartorius Stedim Biotech GmbH) was equipped with a two-stage stirrer with three-bladed segment impellers (*d* = 54 mm) and commonly used reactor installations (i.e., temperature sensor, ring sparger, autoclave tube, sampling tube) as well as an EasyFerm® Plus pH electrode (Hamilton Germany GmbH) and a VisiFerm® DO dissolved-oxygen electrode (Hamilton Germany GmbH). Bioreactor control and documentation were performed via a Biostat B digital control unit (DCU) with the accompanying BioPAT® MFCS/win 4.0 control software (Sartorius Stedim Biotech GmbH). To prevent uneven lighting (reflections or shading) due to sunlight, a dark tent was installed around the experimental setup (see Fig. 1). The bioreactor was positioned in front of an LED panel (Panel Value 1200 × 600 TPB 65 W 4000 K WT from Ledvance GmbH), which was equipped with a constant current supply driven by an Arduino® Due microcontroller (Arduino S.r.l.) to prevent flickering of the backlight. This microcontroller ensured precise synchronization of the backlight activation with the start of the mixing-time experiment.

### Experimental setup with single-use bioreactor

**Fig. 2:**
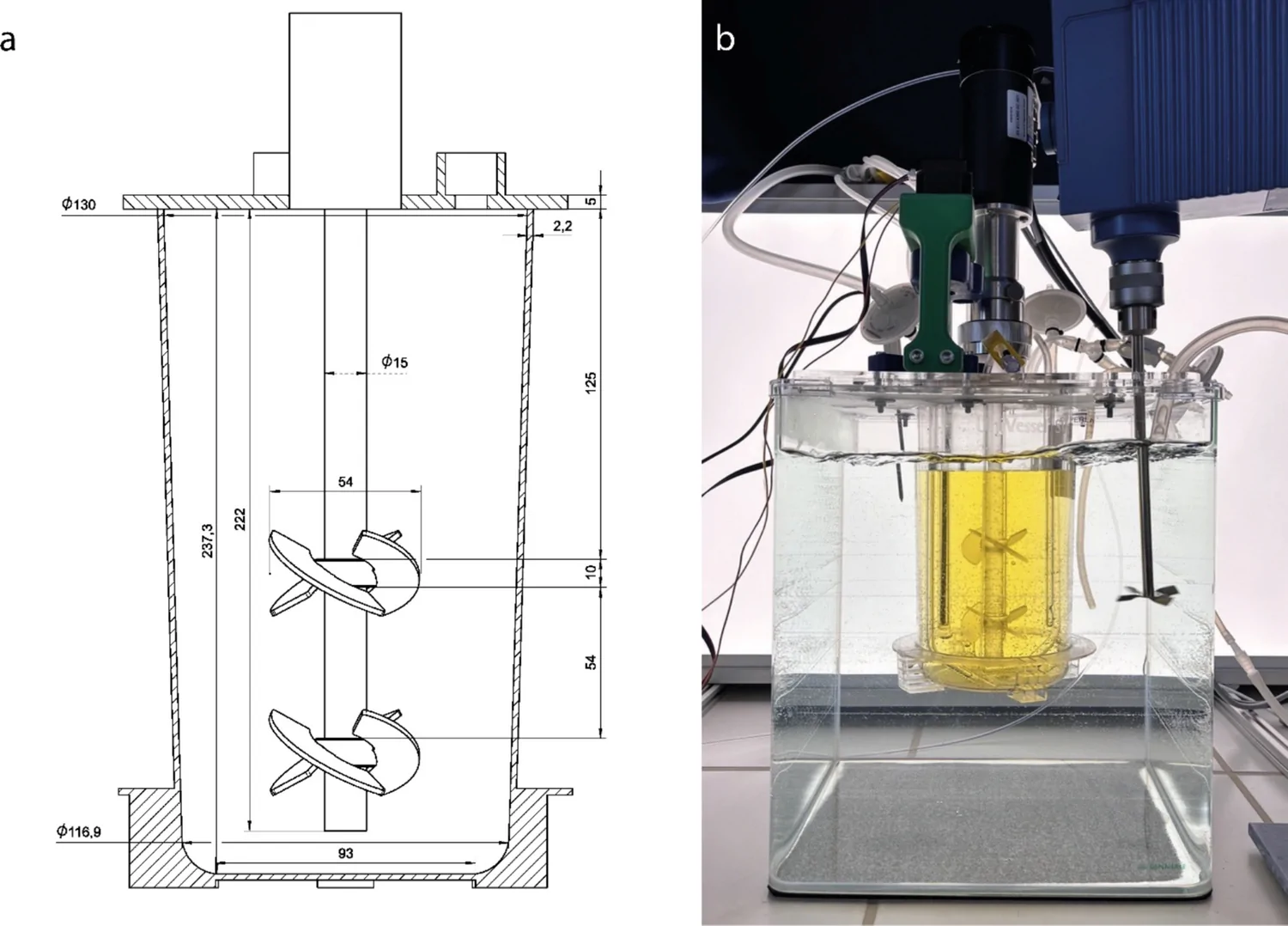
Dimensions of the single-use bioreactor used (**a**) and the single-use bioreactor mounted in an acrylic tank, enabling heat exchange while ensuring transparency for the video recordings (**b**)

A stirred SU bioreactor Univessel® 2 L SU (Sartorius Stedim Biotech GmbH), equipped with a two-stage stirrer with three-bladed segment impellers (equivalent to the glass reactor setup), was used for the mixing-time experiments (see Fig. 2a). An L-type sparger was molded into the plastic vessel, whereas its glass counterpart was equipped with a ring sparger. For the aerated experiments, an air flow rate of 0.5 vvm (which refers to 1 sLpm) was selected and applied continuously throughout each experiment. The temperature control via a tempering jacket was replaced by a water bath to improve optical accessibility and imaging conditions (see “Results”). For this purpose, a Nano Cube® 30 L acrylic tank (Dennerle GmbH) was used. A custom lid was designed for this water bath and precision-milled from a sheet of acrylic glass. The SU bioreactor could be attached to the acrylic glass lid so that its position was maintained in the water bath regardless of the bath’s fill level. Additional openings were added to the lid for supplying and removing thermostat-controlled water, as well as for attaching the linear actuator (see “Automated addition of acid for mixing-time experiments”). A photo of the experimental setup is shown in Fig. 2b. The SU bioreactor was controlled by the same DCU and software as the glass bioreactor and placed within the water bath in the same dark tent in front of the LED panel (see “Experimental setup with glass bioreactor”).

### Automated addition of acid for mixing-time experiments

**Fig. 3:**
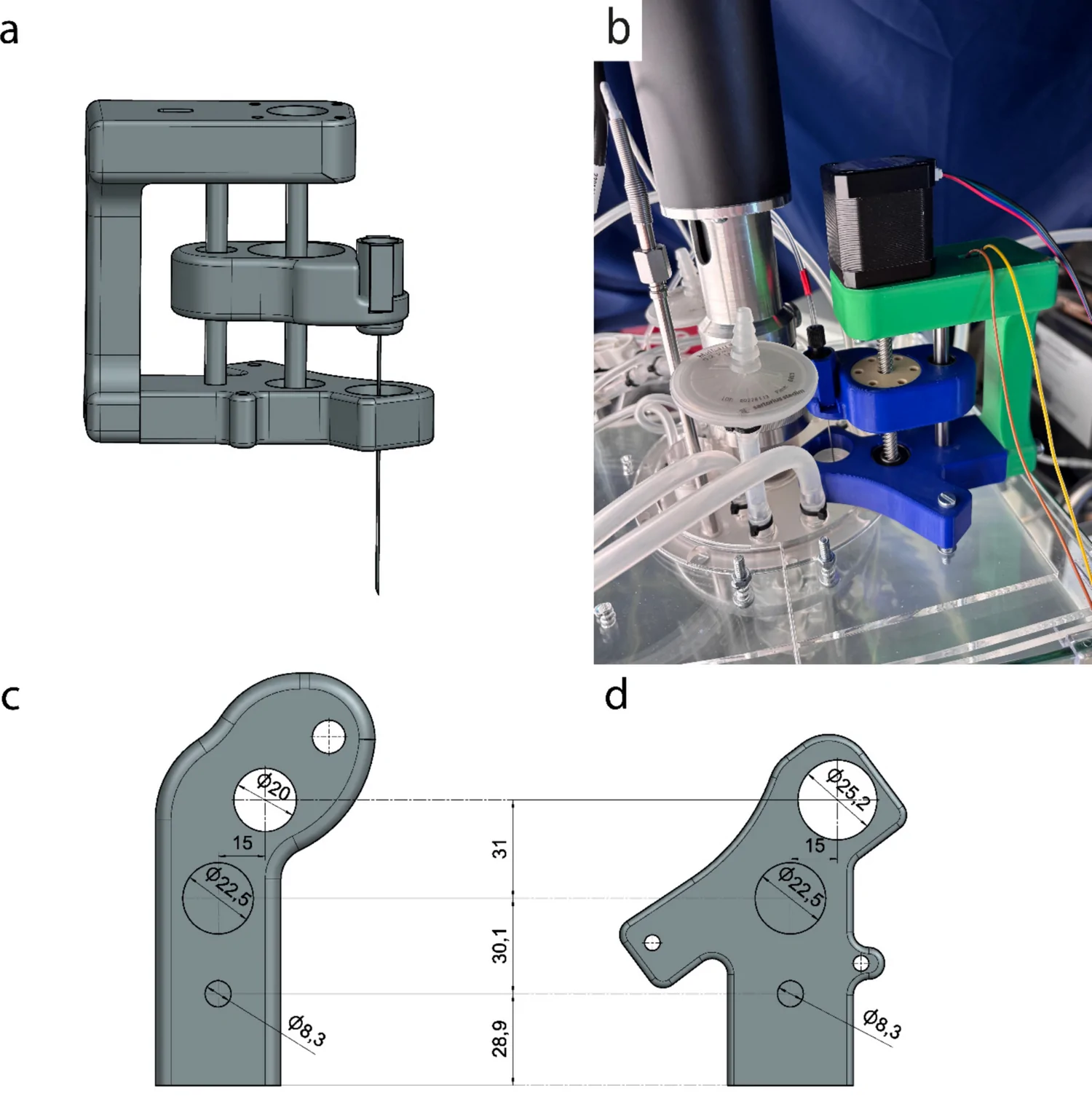
Linear actuator for automated mixing-time experiments; 3D construction in CAD (**a**); assembled linear actuator mounted on the single-use bioreactor (**b**); base plate for Univessel® 2 L DW (**c**); base plate for Univessel® 2 L SU (**d**)

The addition of acid and the activation of the backlight were synchronized in both the manual and automated measurements using an Arduino® Due microcontroller (Arduino S.r.l.) equipped with a trigger button to start the measurement. In this way, the start of the mixing-time experiment was precisely identified in the recorded videos, as the backlight was switched on when the acid was added. Furthermore, a Hamilton Microlab® ML625-DIL Advanced Dual Syringe Diluter (Hamilton Bonaduz AG) with two syringes and a linear actuator for precise positioning of the syringe-pump cannula was integrated into the automated tests. Two syringe combinations were used: 100 µL/10 mL and 1 mL/10 mL. For the standard procedure, the acid (90 µL of 4 M HCl) was added to the system via the 100-µL syringe and a 0.8-mm cannula with an injection speed of 50 µL/s, resulting in a pipetting duration of approximately 1.8 s. This slow addition of acid, as described by Fitschen (2022), was chosen because it prevented the mixing process from being influenced by a high impulse, which could cause macroscopic structures to be advected from the point of injection toward the impeller region. To test the influence of the injection speed, the acid (180 µL of 2 M HCl) was added to the system via a 1-mL syringe at injection speeds of 100 µL/s, 210 µL/s, 440 µL/s, and 650 µL/s, resulting in injection times of 1.8 s, 0.86 s, 0.41 s, and 0.28 s, respectively. The linear actuator was designed to attach the cannula of the syringe pump. This enabled the addition of acid in a reproducible manner in terms of the exact position (immersion height and angle), tracer volume, and injection flow rate of the tracer addition (hereinafter referred to as the “injection speed”). The cannula was positioned in the center of one PG13.5 port of the bioreactor (corresponding to a radial position of *r* = 45 mm from the center of the stirrer shaft). The immersion height was set to 20 mm below the liquid surface, and the immersion angle perpendicular to the liquid surface. The linear actuator was designed using the computer-aided design (CAD) software NX™ 2007 (Siemens AG). The linear actuator comprised a combination of 3D-printed parts and commercially available components such as the spindle and stepper motor (see Fig. 3).

The design included an interchangeable base plate, which enabled the linear actuator to be adapted for use with different bioreactors (see Fig. 3c and d). The two upper parts were designed for mounting a standard stepper motor. The 3D-printed parts were manufactured using a Prusa MK4 or MK3 FDM 3D printer (Prusa Research a.s.) with G-Code generated with PrusaSlicer 2.9.0 software (Prusa Research a.s.). The 3D-printed parts were printed from a glycol-modified polyethylene terephthalate (PETG) filament (PolyMaker GmbH) with 15% gyroid infill and ≥ 4 perimeters to enhance stiffness. A 0.10 mm layer height was selected to improve the fine features. All parts were produced with a brim and model-generated supports to ensure adhesion and overhang quality.

To achieve highly reproducible linear motion, a Nema 17 HE19-2004S bipolar hybrid stepper motor (Stepperonline UG) was used. This cost-effective actuator, which is widely used in desktop additive manufacturing and CNC, provided a 1.8° full-step angle (± 5% step accuracy) and a holding torque of 0.55 Nm. The phase current was set to 2 A with a nominal 12 V supply. Motion control was implemented on an Arduino® Uno microcontroller (Arduino S.r.l.) interfacing with a DRV8825 stepper-motor driver (Texas Instruments Deutschland GmbH), which provided Arduino® compatibility and integrated over-temperature and over-current protection. The components needed for the linear actuator and their corresponding prices are listed in Table 1.

**Table 1:** Components of linear actuator and cost breakdown (net prices)

Component	Cost of materials (€)
Steep threaded spindle	25.91
Threaded nut	11.36
Precision shaft	1.68
Backlash-free elastomer coupling	8.40
Nema 17 stepper motor	10.92
Ball bearings	5.04
Micro switch (limit switch)	1.89
Arduino® Uno	16.22
Expansion board	4.20
DRV8825 stepper-motor driver module	5.87
Jumper wire	5.87
Power supply unit	7.55
Screws	0.84
Threaded inserts	1.31
PETG material costs (~ 200 g)	4.24
Total cost	111.32

The motion of the linear actuator, controlled by the Arduino® microcontroller, was synchronized with the syringe diluter and the Arduino®-controlled LED backlight. The control system workflow was as follows: When the syringe-diluter trigger was activated, the required amount of acid (e.g., 90 µL) was drawn from a reservoir into the cannula using the smaller of the diluter’s two syringe pumps. In addition, 5 mL of water was drawn into the diluter’s second syringe, which was later used to flush the cannula. After connecting the cannula to the linear drive and starting the video recording, a series of processes was initiated when the syringe diluter was manually triggered again. First, the linear actuator moved the cannula below the surface of the liquid. Once the (always identical) end position (measured with an end-stop sensor) was reached, the acid was added (e.g., at a rate of 50 µL/s), and the light source was simultaneously switched on. Next, the cannula was moved out of the liquid again using the linear actuator. At the end of the mixing-time experiment (i.e., after the video recording had finished), the cannula was removed and positioned over a waste container. Pressing the syringe-diluter trigger again initiated the cleaning of the cannula with the water that previously had been drawn in. The entire procedure could also be used to add base in order to restore the initial alkaline conditions in the bioreactor.

## Results

The global and local mixing times *t*_95_ were determined using the colorimetric pH-shift method with bromothymol blue as an indicator, video recordings, and evaluation with a Python script (see “Materials and methods”). Preliminary manual measurements showed substantial differences in the mixing times determined by different individuals using the same experimental parameters. To minimize operator-dependent variability, an SOP was implemented and applied as good practice throughout this work. For the manual measurements, the acid was added through a free PG13.5 port using a mechanical pipette. The pipette tip was intended to be immersed in the liquid at a consistent depth and angle; however, this was more difficult for some operators than for others. Furthermore, maintaining the exact addition speed was difficult, not only when comparing different operators but also for the same operator. Therefore, to increase reproducibility, an automated system was developed and tested that uses a linear actuator and control system (see “Automated addition of acid for mixing-time experiments”).

To prevent inaccuracies in optical measurements associated with the less transparent (milky) wall and the opaque heating/cooling jackets of the SU bioreactors, the SU bioreactor was immersed in a thermostatic, transparent water bath (see “Experimental setup with single-use bioreactor”). The SU bioreactor was attached to the lid and positioned between the acrylic panels so that it floated in the water. This eliminated the need for the stand, which would otherwise hide the lower part of the bioreactor. Consequently, the visible area expanded to the full height of the bioreactor. The front wall of the water bath was aligned perpendicularly to the optical axis of the camera system. The contact between the tempering water and the outer reactor wall increased the plastic’s transparency. In addition, the rectangular water bath eliminated reflections and distortions caused by the curved bioreactor wall without a water bath. These modifications significantly improved the video quality and enhanced the robustness of the image processing. However, the improvement introduced a challenge for the manual measurements, as it became more difficult to immerse the pipette tip at a consistent depth and angle into the liquid in the bioreactor. This was due to optical distortion of the pipette tip caused by the water level in the surrounding water bath. Consequently, additional variability was introduced, even for the same operator. By using the automated process (see “Automated addition of acid for mixing-time experiments”), these challenges were overcome, enabling reproducible results and reliable characterizations of the mixing-time behavior of the SU bioreactor used.

### Comparison of mixing times for manual versus automated measurements

**Fig. 4:**
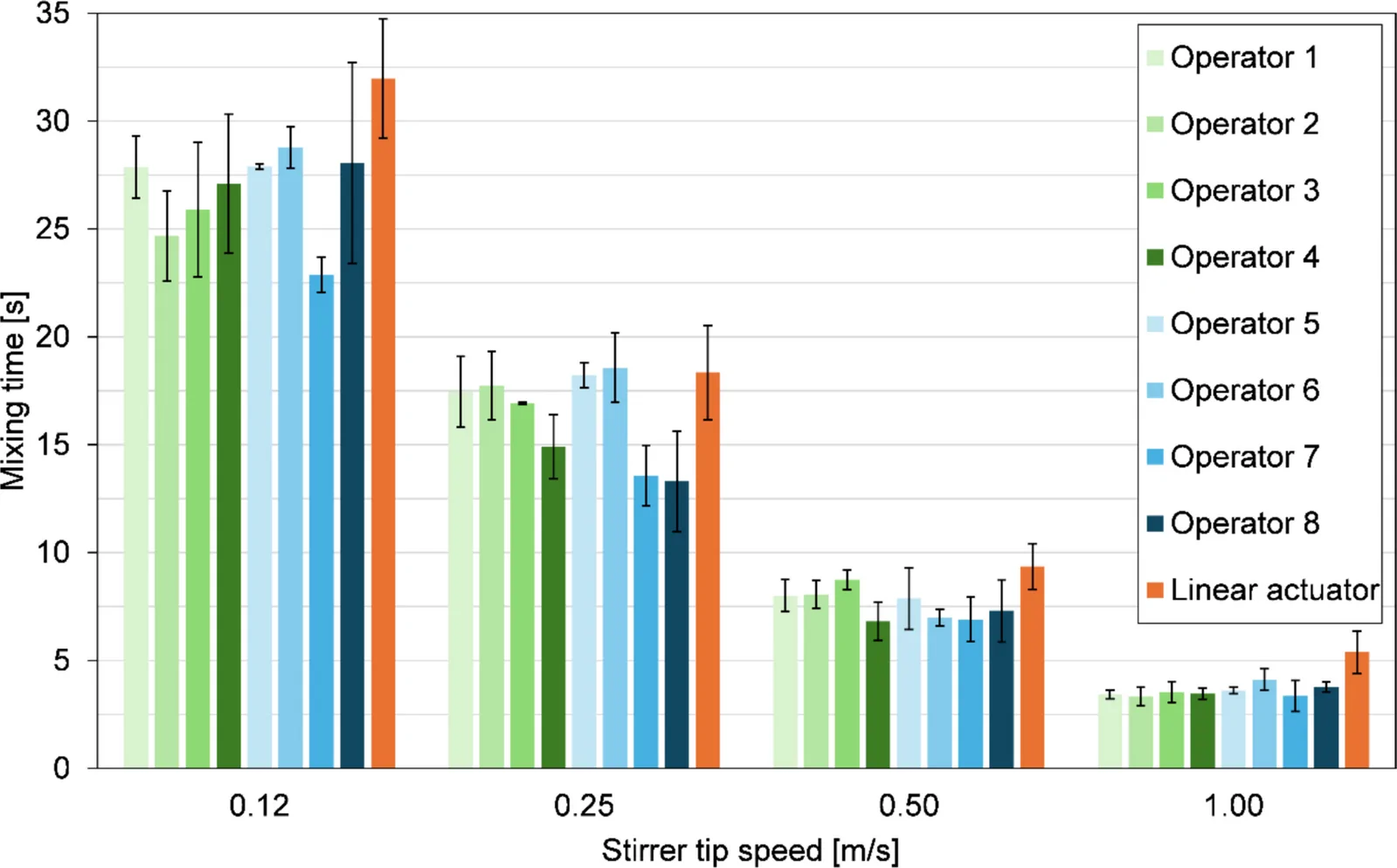
Average of global mixing times *t*_95_ for manual and automated measurements at different stirrer tip speeds; for the manual measurements, each operator completed three replicates at each stirrer tip speed; for automated measurements using the linear actuator, 24 replicates were performed at each stirrer tip speed

To compare the mixing times of the manual measurement with those of the automated measurement, experiments were performed at four different stirrer tip speeds (0.12 m/s, 0.25 m/s, 0.50 m/s, and 1.00 m/s) in the SU setup. The mixing times for the manual measurements were calculated from triplicate measurements performed by eight operators at each stirrer tip speed using a mechanical pipette (P). The mixing times for the automated measurements were calculated from 24 replicates performed at each stirrer tip speed using the linear actuator (LA). The resulting average mixing time of each operator, as well as the average mixing time for the automated measurements, is shown in Fig. 4 and Table 2.

**Table 2:** Comparison of manual and automated measurements to determine the average global mixing times (*t*_95_), as well as the coefficient of variation at different stirrer tip speeds

Stirrer tip speed (m/s)	Average mixing time t_95_ (s)		Coefficient of variation of *t*_95_ (-)	
	Manual	Automated with linear actuator	Manual	Automated with linear actuator
0.12	26.64 ± 2.83	31.97 ± 2.77	0.11	0.09
0.25	16.34 ± 2.36	18.34 ± 2.19	0.14	0.12
0.50	7.59 ± 1.03	9.35 ± 1.06	0.14	0.11
1.00	3.58 ± 0.41	5.39 ± 0.98	0.11	0.18

In some cases, the mixing times determined by different operators showed substantial variability despite the use of an SOP and standardized software-based evaluation (see “Materials and methods”). This poses a major challenge, particularly when mixing-time experiments are used to validate CFD simulations. An analysis of operator-specific replicates revealed differences in reproducibility between individuals (see Fig. 4), likely reflecting variations in experimental experience.

The coefficient of variation (CV), a standardized measure of dispersion, is defined as the ratio of the standard deviation to the mean. The CV thus reflects measurement precision. To enable the consistent comparison of variability across different mixing times, the CV in this study was calculated for each measurement (see Table 2). The average CV values for the manual measurements and those for the automated measurements were identical (CV_avg_ = 0.125), indicating no difference in measurement precision. This result corresponds well with the results of an additional two-sided *F*-test on the residuals (Sachs 2004), which showed no significant difference in measurement variance between the manual and automated measurements (the P/LA variance ratio was 1.02, 95% CI [0.68, 1.53], *p* = 0.931). However, a completely different picture emerges when the test series at a stirrer tip speed of 1.00 m/s is excluded (see Table 2). This aspect is discussed in more detail below.

The mixing times achieved with the linear actuator were longer in almost all cases compared with those determined in the manual measurements (see Fig. 4 and Table 2). On average, the mixing times were 1.8 s (at stirrer tip speeds of 0.50 m/s and 1.00 m/s), 2.0 s (at 0.25 m/s), and 5.3 s (at 0.1 m/s) longer for the linear actuator. To test whether the average mixing times of the manual measurements were significantly lower than those of the automated measurements, a pooled-variance (equal variances) *t*-test (Gosset 1908; Hogg et al. 2020) assuming equal variances was performed. Because the one-tailed *p*-value was *p* = 0.0276, it was concluded that the average mixing time of the manual measurements was significantly lower than that of the automated measurements at the *α* = 0.05 significance level. The effect was mainly due to the injection speed of 50 µL/s (specified in the SOP), which resulted in an injection time of 1.8 s. On average, the eight operators added the acid within just 0.41 ± 0.19 s, which was more than four times as fast as specified. Despite the use of an SOP, the specified settings were not implemented in the same way by each operator. A comparison of the absolute injection times showed that with a minimum of 0.12 s and a maximum of 1.18 s, corresponding to an injection time of 0.6% to 32.1% of the global mixing time *t*_95_, the observed fluctuations are directly attributable to this. None of the operators maintained the injection speed specified in the SOP.

**Fig. 5:**
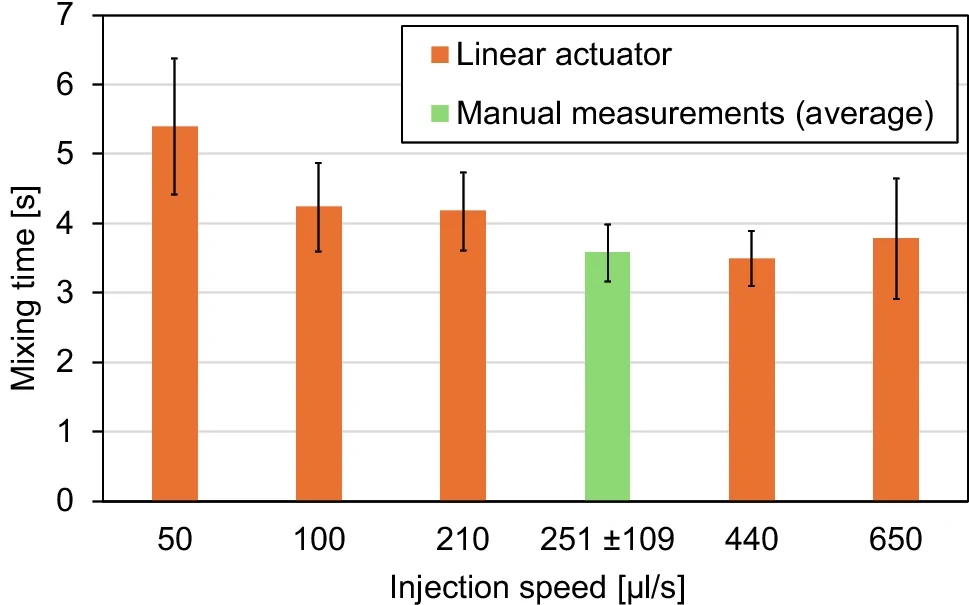
Average of global mixing times *t*_95_ for manual and automated measurements resulting from different injection speeds

The injection time of the linear actuator was 6% (at a stirrer tip speed of 0.12 m/s), 10% (at 0.25 m/s), 19% (at 0.50 m/s), and 33% (at 1.00 m/s) of the global mixing time *t*_95_. This percentage of injection time relative to mixing time resulted in significantly higher values than the 1% proposed by Rautenbach et al. (2026), especially at the higher stirrer tip speed. Notably, the injection speed specified in the SOP was originally used for mixing times of up to 47 ± 17 s, with the injection time corresponding to approximately 4% of the global mixing time (Fitschen et al. 2021). To investigate the influence of the injection time (and thus the injection speed) on the mixing-time experiments, a new set of automated measurements with higher injection speeds was completed for the highest stirrer tip speed (i.e., 1.00 m/s). To achieve this, a larger syringe was used (1 mL instead of 100 µL). Using this syringe created a limitation because the 1 mL syringe was not suitable for accurately dispensing a volume of 90 µL. Therefore, the injection volume was increased to 180 µL. Accordingly, a suitable acid concentration of 2 M HCl was selected. In total, four injection speeds (100 µL/s, 210 µL/s, 440 µL/s, and 650 µL/s) were used with the linear actuator to determine the global mixing times. The results are shown in Fig. 5.

The injection speed of 100 µL/s for a 180 µL volume resulted in an injection duration of 1.8 s, which mirrors the previously used injection time of 1.8 s at 50 µL/s and 90 µL. The CV values obtained with an injection volume of 180 µL were 0.18 (at 50 µL/s), 0.15 (at 100 µL/s), and 0.14 (at 210 µL/s). It was concluded that higher injection speeds improved the precision, as the injection speed decreased the CV value. A further increase of the injection speed to 440 µL/s supported this trend, as the CV dropped to 0.11. At an injection speed of 440 µL/s for a volume of 180 µL, the resulting injection time was 0.41 s. This value was consistent with the average injection time of 0.41 s observed for the manual measurements performed according to the SOP. A comparison of both sample sets showed that, at an identical injection time of 0.41 s, the automated measurements (*t*_95_ = 3.50 ± 0.40 s) resulted in mixing times comparable to those obtained with the manual measurements (*t*_95_ = 3.58 ± 0.41 s) (see Fig. 5).

In addition, a correlation analysis was performed between the CV values and the percentage of injection times for all 96 manual and 144 automated measurements. This analysis revealed that the percentage of injection time had no measurable influence on the CV values (data not shown). This finding suggests that other parameters may have a more pronounced effect on the CV value.

As mentioned above, the CV values measured at an injection speed of 50 µL/s for a 90 µL volume were lower in the automated measurements than in the manual measurements at the three lower stirrer tip speeds of 0.12 m/s, 0.25 m/s, and 0.50 m/s (see Table 2). Only at a stirrer tip speed of 1.00 m/s was a contrary effect observed due to the high percentage of the injection time in relation to the total mixing time. This problem was solved by adjusting the injection speed to 440 µL/s (with a volume of 180 µL), which resulted in CV values equal to those obtained in the manual measurements. This adjustment reduced the overall average CV for the automated measurements to 0.108, representing a 13.6% reduction from the average CV of 0.125 for the manual measurements. Thus, it was concluded that the automated experiments significantly increased the precision of the mixing-time experiments due to the consistent test parameters, particularly the constant injection speed, in addition to the controlled immersion depth, immersion angle, and tracer volume.

### Spatially resolved analyses of mixing in SU bioreactors

**Fig. 6:**
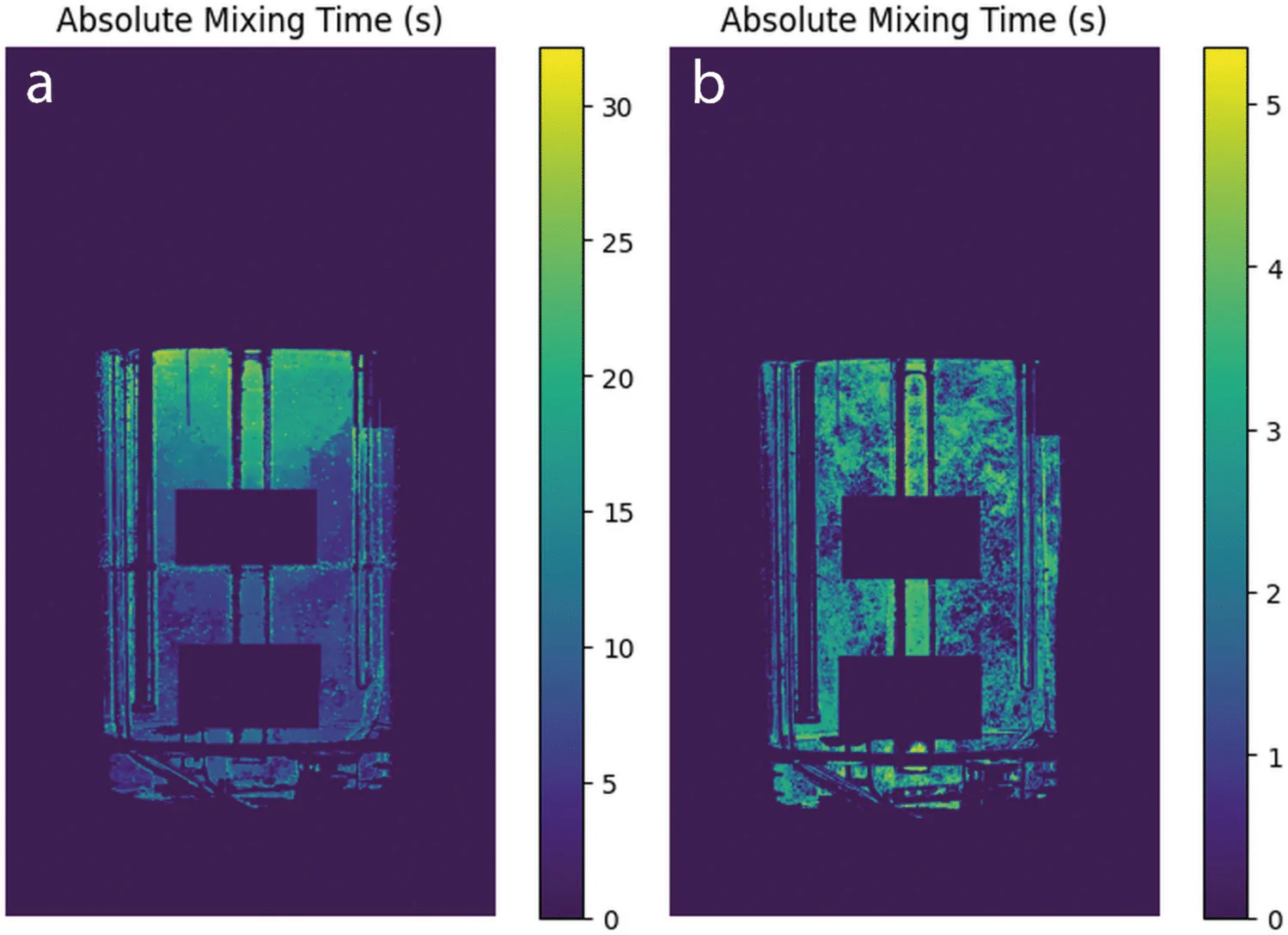
2D color-coded diagram generated by the Python script (Barth et al. 2026) showing the local mixing-time distribution in the stirred single-use bioreactor with low stirrer speed (**a**) and high stirrer speed (**b**)

The spatially resolved analyses of the SU bioreactor (see Fig. 6) showed a variation of the local mixing throughout the reaction vessel. In both methods (manual and automated), variations in the local mixing time were observed. In all experiments, low stirrer tip speeds resulted in a strong segmentation of the bioreactor into two zones, creating a separation layer above the two impellers of the two-stage stirrer. A video analysis demonstrated that the lower area in the bioreactor mixed first, followed by a delay before the upper zone of the bioreactor began to change the color. This was also evident in the mixing-time distribution, which exhibited a bimodal distribution. The separation between the upper and lower mixing zones was also clearly visible in the 2D color-coded diagram (see Fig. 6a). At higher speeds, this effect of zone formation decreased significantly due to the rapid overall mixing (see Fig. 6b).

### Comparison of SU and glass bioreactors

**Fig. 7:**
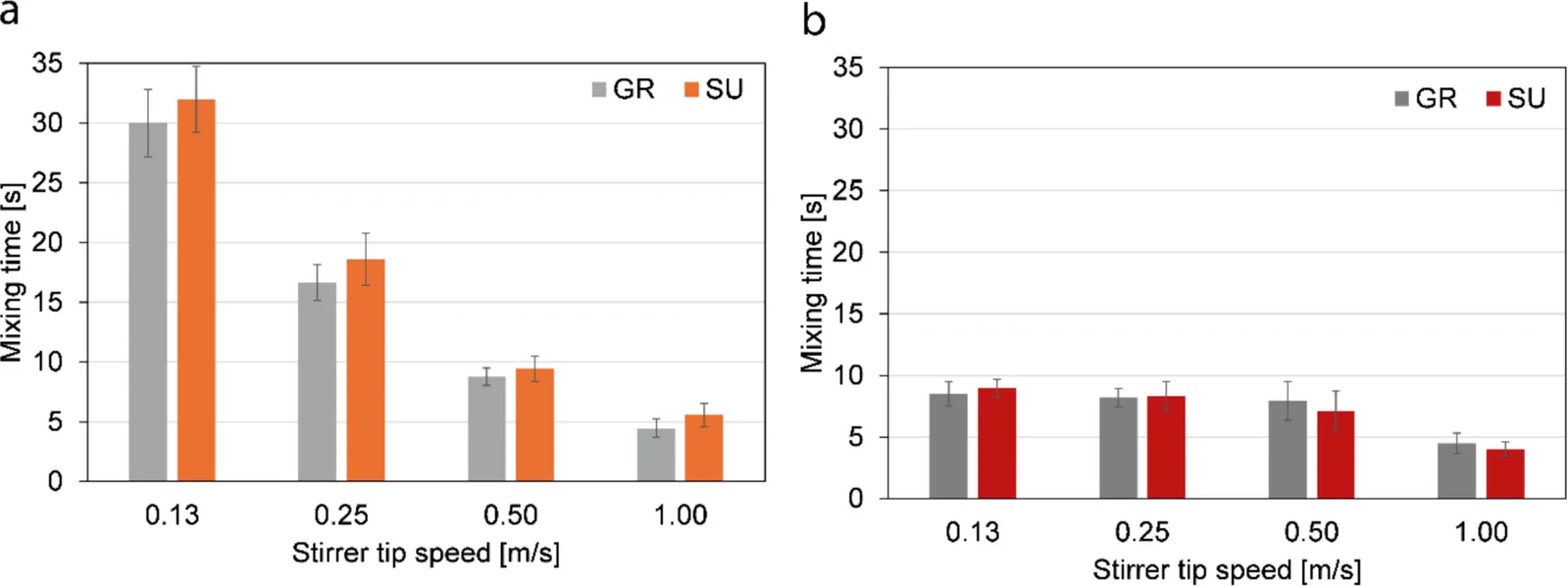
Mixing time in single-use (SU) and glass (GR) bioreactors at various stirrer tip speeds under non-aerated conditions (**a**) and with 1 sLpm aeration (**b**)

The mixing times of the Univessel® 2 L SU bioreactor were compared with those of the Univessel® 2 L DW glass bioreactor (GR) at various stirrer tip speeds (0.125 m/s, 0.25 m/s, 0.50 m/s, and 1 m/s) under non-aerated and aerated (1 sLpm, 0.5 vvm) conditions (see Fig. 7). For this comparison, automated measurements with the linear actuator were used due to their better reproducibility. In the non-aerated condition (see Fig. 7a), the replicate variance did not differ significantly between the two bioreactors (two-sided *F*-test on residuals: GR/SU variance ratio was 0.72, 95% CI [0.45, 1.22], *p* = 0.226). Under the aerated conditions (see Fig. 7b), the replicate variance also did not differ between the two bioreactors (two-sided *F*-test on residuals: GR/SU variance ratio was 0.98, 95% CI [0.55, 1.74], *p* = 0.939).

To test whether the reaction vessel type affected the mixing time, a one-sided hypothesis (SU bioreactor > glass bioreactor) was pre-specified. A pooled-variance *t*-test (Gosset 1908; Hogg et al. 2020) gave *p* = 0.187 for the non-aerated conditions and *p* = 0.354 for the aerated condition, providing no evidence that mixing times were higher in the Univessel® 2 L SU bioreactor than the Univessel® 2 L DW glass bioreactor.

Aeration drastically reduced the mixing times compared with the non-aerated conditions for both bioreactors at lower stirrer tip speeds (0.13 m/s, 0.25 m/s). This reduction in mixing time was attributed to additional momentum input from the gas bubbles and the transition to a combined bubble- and stirrer-driven flow regime. For the higher stirrer tip speeds of 0.50 m/s and 1 m/s, this effect was not observed in either bioreactor, where the mixing times were similar for the aerated and non-aerated conditions. In this case, the power input through the gas bubbles was negligible compared with the power input introduced by the stirrer.

## Discussion

Every mixing process in a bioreactor is subject to statistical fluctuations that arise from minor differences in the flow paths within the flow field. These variations occur spontaneously and are typically not observable at the macroscopic level. As a result, mixing-time experiments, whether performed manually or automatically, inherently exhibit statistical variability. Therefore, all mixing-time experiments should be conducted with a sufficient number of replicates and analyzed statistically (Rautenbach et al. 2026). Accordingly, it is not surprising that, in the present study, the mixing times remained statistically distributed with a corresponding standard deviation, even when using the linear actuator.

In addition to the inherent fluctuations of this type of experiment, further deviations in measured mixing times can arise if operators do not perform the experiments consistently. Factors such as the horizontal position of the tracer addition point (i.e., radial distance from the agitator shaft, proximity to electrodes and other reactor internals), immersion depth, immersion angle, tracer volume, and injection speed all play an important role. In practice, no single “true” mixing time exists. If the injection speed is too low, longer mixing times are observed even though the flow field remains unchanged. If the injection speed is too high, an additional impulse is introduced into the liquid, altering the flow field (Fitschen 2022). This is supported by the results obtained at the highest injection speed (650 µL/s), which led to a marked increase in the CV value from 0.14 to 0.23, indicating a significant reduction in precision. This finding suggests that excessively high injection speeds introduce a significant impulse that perturbs the flow field beyond the injection site. Indeed, Hartmann et al. (2006) described this phenomenon as the relative importance of jet mixing and stirred mixing with high injection speeds. To overcome this problem, Distelhoff et al. (1997) limited the tracer injection to 1% of a typical mixing time, while Hartmann et al. (2006) set the injector speed to correspond to half an impeller revolution. Nevertheless, manually determined mixing times can vary substantially between operators. In the present research, this applied even though an SOP was used, as the required settings could not be implemented consistently by all operators.

In addition to the experimental implementation, the evaluation of the measurement data plays a key role. Different perceptions of colors or brightness levels in tracer experiments with pH-sensitive color indicators make objective evaluations difficult or, in some cases, impossible. Using software-supported, automated evaluation helps to solve this problem and obtain results that are, in principle, independent of the persons involved. However, software-supported, automated evaluation cannot prevent experimental deviations and, therefore, cannot eliminate experimental variability.

In the experiments that used the SOP, the average mixing time for the manual measurements was significantly shorter than for the automated experiments. This was attributed to the slow (but steady) addition of acid at a constant injection speed in the automated cases. This slow acid addition also increased the precision of the measurements at the three lower stirrer tip speeds (0.12 m/s, 0.25 m/s, and 0.50 m/s) but not at 1.00 m/s. At a stirrer tip speed of 1.00 m/s, improved precision was achieved after adjusting the injection speed (see “Comparison of mixing times for manual versus automated measurements”). The overall CV of the mixing-time experiments could be improved by 13.6% through the automation approach.

To avoid the influence of injection speed, an alternative approach involves setting up an automation system using only a syringe diluter or syringe pump without a linear actuator, which ensures precise control of the injection speed during tracer addition and eliminates this source of variability. However, the problem remains that the operator must immerse the cannula of the syringe pump into the liquid in the bioreactor at the same depth and angle. Thus, the linear actuator used in the present research effectively improves the experiment’s reproducibility, and at minimal additional cost if a remote-controlled syringe diluter or syringe pump is available.

In summary, the automation approach reduced inter-operator variability and improved the reproducibility of both the global and local mixing-time estimates. This approach facilitates robust bioreactor characterization and the identification of dead zones, and it supports the analysis of shear exposure and flow-field features relevant to shear-sensitive cultures and CFD validation. When matching a mixing-time experiment with the corresponding CFD simulation, it is important to ensure that the experimental parameters, particularly the injection speed, are identical in both procedures. Only under these conditions can CFD simulations be reliably validated.

This work has demonstrated the feasibility of evaluating both the global mixing time and the distribution of local mixing times of stirred SU bioreactors with acceptable reproducibility using the proposed experimental setup with the linear actuator (see “Spatially resolved analyses of mixing in SU bioreactors”). The two bioreactors that were investigated, Univessel® 2 L DW and Univessel® 2 L SU, were geometrically similar. As expected, both bioreactors exhibited similar mixing-time behavior. This similarity was confirmed by the automated experiments and the resulting reproducibility (see “Comparison of SU and glass bioreactors”). Although additional modifications may be required depending on the geometry, this method can also be applied to other bioreactor geometries. However, visual access must be ensured.

## Data Availability

The authors declare that the data supporting the findings of this study are available within the paper. Should any raw data files be needed in another format they are available from the corresponding author upon reasonable request.

## Abbreviations

CAD: Computer-aided design
CFD: Computational fluid dynamics
CV: Coefficient of variation
DW: Double-walled
LA: Linear actuator
LED: Light-emitting diode
P: Pipette (refers to manual measurements)
PETG: Glycol-modified polyethylene terephthalate
ROI: Region of interest
SOP: Standard operating procedure
sLpm: Standard liters per minute
SU: Single-use
*t*_95_: Time at which the deviation of *θ*(*t*) from its final value falls within a tolerance of *ε* = 0.05
vvm: Volume of gas per volume of liquid per minute
*ε*: Tolerance
*θ*(*t*): Spatially averaged dimensionless local concentration
*θ*(*r*,*t*): Dimensionless local concentration at position *r* and time *t*

## Declarations

Both the base plate of the linear actuator and the acrylic glass lid can be adapted to different single-use bioreactors. Design files are available from the corresponding author upon reasonable request. Please contact the corresponding author for this purpose.
